# Internal mammary lymph nodes radiotherapy of breast cancer in the era of individualized medicine

**DOI:** 10.18632/oncotarget.20186

**Published:** 2017-08-11

**Authors:** Bin-Bin Cong, Xiao-Shan Cao, Lu Cao, Hui Zhu, Yi-Shan Yu, Jin-Ming Yu, Yong-Sheng Wang

**Affiliations:** ^1^ School of Medicine and Life Sciences, University of Jinan and Shandong Academy of Medical Sciences, Jinan, Shandong, 250200, China; ^2^ Breast Cancer Center, Shandong Cancer Hospital Affiliated to Shandong University, Jinan, Shandong, 250117, China; ^3^ Shandong Academy of Medical Sciences, Jinan, Shandong, 250062, China; ^4^ Department of Radiation Oncology, Ruijin Hospital, Shanghai Jiaotong University School of Medicine, Shanghai, 200025, China; ^5^ Department of Radiotherapy, Shandong Cancer Hospital Affiliated to Shandong University, Jinan, Shandong, 250117, China

**Keywords:** breast cancer, internal mammary lymph nodes, sentinel lymph node biopsy, internal mammary lymph nodes irradiation

## Abstract

Inclusion internal mammary lymph nodes as a part of regional nodal irradiation have a potential to reduce local recurrence, distant recurrence, and improve survival in breast cancer. However, the increased risk of cardiac toxicity and lungs injure associated with internal mammary lymph nodes irradiation has drew more and more attention. Estimating risk of metastasis in internal mammary lymph nodes based on axillary lymph nodes metastasis status is not always reliable: low-risk do not always mean negative in internal mammary lymph nodes and high-risk do not always indicate positive in internal mammary lymph nodes. Inaccurate prediction of in internal mammary lymph nodes metastasis might lead to over- or under-treatment of in internal mammary lymph node. Internal mammary sentinel lymph node biopsy is a minimally invasive technique which has a high potential to accurately evaluate the metastasis status in in internal mammary lymph nodes and improve accuracy of nodal staging. This technique might be a useful tool to guide individualized internal mammary lymph nodes irradiation.

## INTRODUCTION

Internal mammary lymph nodes (IMN) metastasis has a similar prognostic importance as axillary lymph nodes (ALN) involvement in breast cancer patients. IMN metastases have been demonstrated to occur in 28–52% of ALN positive patients and 5–17% of ALN negative patients [1, 2]. Regional nodal irradiation (RNI), together with chest-wall or conserved breast irradiation, has been proved to not only improve local control, but also reduce any recurrence and increase overall survival in high-risk breast cancer patients. Even if the evidence supporting delivery of RNI in patients with positive ALN is increasing, and National Comprehensive Cancer Network (NCCN) Breast Cancer Clinical Practice Guidelines updated in 2016 has raised the level of recommendation for internal mammary lymph nodes irradiation (IMNI), value of IMNI remains more controversial than other sites of RNI. Hesitations in delivering about IMNI are listed as follow: rate of clinically detected IMN recurrence is low, studies evaluating separately the role of IMNI are scared and inclusion of IMN as a part of RNI would increase dose-volume of cardiac and pulmonary irradiation. Current review aims to evaluate the role of IMNI based on available trials, and to integrate diagnostic and surgical procedure as to identify the subset of patients who might benefit more from IMNI.

### Benefit of internal mammary lymph node irradiation

#### Survival benefit of internal mammary lymph nodes irradiation

For most breast cancer patients, improvement of systemic therapy has significantly decreased the risk of death from distant metastasis, after which the optimized local therapy could, eventually, contribute more to improving survival in the era of molecular subtypes guided adjuvant system therapy [3]. Meta-analysis from Early Breast Cancer Trialists’ Collaborative Group found that post-mastectomy radiotherapy (PMRT) given to chest wall irradiation and regional nodes could significantly reduce locoregional recurrence, overall recurrence, and breast cancer mortality in women with node positive disease. About one breast cancer death avoided in 20 years after PMRT were of comparable relevance to every 1.5 recurrences of any type (i.e., either loco-regional or distant) avoided during the first 10 years [4]. The MA.20 trial from NCIC Clinical Trials Group found that addition of RNI (including IMNI and irradiation to axilla in those not fully dissected) to whole breast irradiation reduced the rate of breast cancer recurrence in node-positive and high-risk node-negative breast cancer patients. After 10-year follow-up, the rate of disease free survival (DFS) in RNI group was significantly improved than that in the control group (82.0% vs. 77.0%; [HR] 0.76 [95% CI, 0.61–0.94], *P* = 0.01). The rate of distant disease free survival (DDFS) was also improved in the RNI group (86.3% vs. 82.4%; [HR] 0.76 [95% CI, 0.60–0.97]; *P* = 0.03). In the subset of patients with estrogen receptor negative disease, RNI even improved rate of overall survival (OS) with a difference approaching statistical significance (81.3% vs. 73.9%, [HR] 0.69 [95% CI, 0.47 to 1.00]; *P* = 0.05) [5]. EORTC 22922/10925 study, similarly evaluating value of addition of RNI (including IMNI) to chest wall or whole breast irradiation, showed that RNI significantly improved DFS (72.1% vs. 69.1%, [HR], 0.89 [95% CI, 0.80–1.00], *P* = 0.04), DDFS (78.0% vs. 75.0%, [HR], 0.86 [95% CI, 0.76–0.98], *P* = 0.02), and reduced breast cancer mortality (12.5% vs. 14.4%, [HR], 0.82 [95% CI, 0.70–0.97], *P* = 0.02) [6]. RNI was also demonstrated to increase OS, although without statistical significance (OS, 82.3% vs. 80.7%, [HR] 0.87 [95% CI, 0.76–1.00], *P* = 0.06). Thus, it is solid enough to prove that addition of RNI, with IMNI as a constant component, contributed to a reduction in loco-regional recurrence and distant metastasis in node positive and high-risk node negative patients, with a trend of improvement in survival in selected sub-population.

As to extrapolate role of IMNI from the above two randomized trials, the major limitation is that it is very difficult to differentiate the contribution of IMNI apart from RNI as a whole. In an early nonrandomized prospective trial, Stemmer et al evaluated the role of IMNI in high-risk stage II–IIIA breast cancer patients. IMNI was performed on 67 out of 100 patients with a total dose of 50.4 Gy using an anterior electron field. Significant improvement in DFS was observed with IMNI (73% vs. 52%; *P* = 0.02), with a trend towards improved OS (78% vs. 64%; *P* = 0.08) after a median follow up of 6.4 years [7]. Until now, there was only one randomized trial evaluating the value of IMNI in addition to chest-wall and supraclavicular nodes irradiation. In this French trial, after 11.3-year median follow-up, no benefit on OS was demonstrated in the IMNI group (59.3% vs. 62.6%, *P* = 0.8). Overestimation of absolute improvement of OS (10% of difference at 10 years) and the risk of IMN involvement (nearly 25%) probably undermined the power of this study to detect difference in OS and lead to negative result [8].

In the prospective cohort study of DBCG-IMN, 1,492 patients with right-sided breast cancer received IMNI and remaining 1,597 patients with left-sided breast cancer did not receive IMNI. All patients had positive ALN. After 8-year follow-up, OS in the IMNI group was significantly improved (75.9% [95% CI, 73.6–78.0] vs. 72.2% [95% CI, 69.9–74.4]; [HR] 0.82 [95% CI, 0.72–0.94], *P* = 0.005), breast cancer mortality rate was significantly reduced (20.9% [95% CI, 18.8–23.0] vs. 23.4% [95% CI, 21.3–25.5]; [HR] 0.85 [95% CI, 0.73–0.98], *P* = 0.03) [9]. Ten percent of patients with left-sided breast cancer also received IMNI, which represent that the population had more positive ALN and medial or central tumor.

It is worth noting that additional RNI including IMN was found to improve DFS, DDFS, and OS in stage I–III breast cancer in a meta-analysis of the MA.20, EORTC22922/10925, and French trials. The absolute benefits in OS were 1% in the MA.20 trial at 10 years, 1.6% in the EORTC22922/10925 trial at 10 years, and 3.3% in the French trial at 10 years (all *P* < 0.05), but no significance found in every single trial. RNI in the MA.20 and EORTC22922/10925 trial was associated with a significant improvement of DFS (HR 0.86 [95% CI, 0.78–0.95]) and DDFS (HR 0.84 [95% CI 0.75–0.94]) [10].

The characteristics of these RNI studies mentioned above are summarized in Table 1.

**Table 1:** Summary of the regional lymph nodes irradiation studies

	Whelan *et al*.^[5]^, 2015	Poortmans *et al*.^[6]^, 2015	Hennequin *et al*.^[8]^, 2013	Stemmer *et al*.^[7]^, 2003	Thorsen *et al*.^[9]^, 2016
**Study Types**	Randomized	Randomized	Randomized	Prospective Nonrandomized	Prospective Nonrandomized
**Enrolling Year**	2000–2007	1996–2004	1991–1997	1994–1998	2003–2007
**Eligible Criteria**	ALN+/ High-risk ALN-	ALN+/ Central or medial tumor	ALN+/ Central or medial tumor	Stage II-IIIA	ALN+ Left-side IMNI Right-side no IMNI
**No. of pts**	1,832	4,004	1,334	100	3,089
**Median age (year)**	54	54	57	45	56
**Breast Surgery**	100% Lumpectomy	76.1% Lumpectomy 23.9% Mastectomy	100% Mastectomy	54% Lumpectomy 46% Mastectomy	65% Mastectomy 35% Lumpectomy
**Systemic Treatment**	CT (91%); HT (76%)	CT (25%); HT (30%); CT+HT (30%)	CT (61%); HT (52%)	CT (100%); HT (42%)	CT (19%); CT+HT (47%)
**ALN Status**					
**N0**	9.7%	44.4%	24.8%	0%	0%
**N1–3+**	85.0%	43.1%	44.1%	0%	58.9%
**N ≥ 4+**	5.3%	12.5%	31.1%	100%	41.1%
**Target Range**	Breast ± RLN	Breast/chest wall ± RLN	Chest wall + SCV LN ± IMN	Breast/chest wall + SCV LN ± IMN	Breast/chest wall + SCV LN ± IMN
**Intercostal Spaces of IMNI**	1–3	1–5 (LIQ) 1–3 (other)	1–5	1–5	1–4
**Dose of Breast/Chest wall**	50 Gy/25 fx	50 Gy/25 fx	Base on the center	50.4 Gy/28 fx	48 Gy/24 fx
**Dose of IMNI**	45 Gy/25 fx	50 Gy/25 fx	45 Gy/25 fx	50.4 Gy/28 fx	48 Gy/24 fx
**Median FU**	9.5	10.9	11.3	6.4	8.9
**OS % Improved**	1%, *P* = 0.38	1.6%, *P* = 0.06	3.3%, *P* = 0.8	14%, *P* = 0.8	3.7%, *P* = 0.005
**Other Outcomes Improved**	DFS 5%, *P*= 0.01; BCM 1%, *P*= 0.11	DFS 3%, *P*= 0.04; DMFS 3%,*P* = 0.02	DFS 3.3%, *P* = 0.35	DFS 21%, *P* = 0.02	BCM 2.5%, *P* = 0.03; DRR 2.3%, *P* = 0.07

Abbreviations: ALN Axillary lymph node; IMNI Internal mammary lymph node irradiation; CT chemotherapy; HT hormonal therapy; RLN Regional lymph node; SCV LN supraclavicular lymph node; IMN Internal mammary lymph node; LIQ lower inner quadrant; fx fractions; FU Following-up; OS overall survival; DFS disease free survival; BCM breast cancer mortality; DMFS distant recurrence free survival; DR distant recurrence rate

#### Guidelines changed in internal mammary lymph nodes irradiation

From 2012 to 2015, the NCCN Breast Cancer Clinical Practice Guidelines constantly recommended IMNI for patients with ALN metastasis after lumpectomy or mastectomy as “strongly consideration” (category 2B). Subsequent to the results of the IMNI trials published, since 2016, NCCN Guidelines have updated recommendation of IMNI for patients with ≥ 4 positive ALNs as category 1, and strongly consider IMNI for patients with 1–3 positive ALNs (category 2A), both after mastectomy and lumpectomy.

The ASCO-ASTRO-SSO panel also updated the ASCO guideline of postmastectomy radiotherapy which recommends treatment generally be administered to both the IMNs and the supraclavicular-axillary apical nodes in addition to the chest wall or reconstructed breast for patients with positive ALNs [11].

Nevertheless, controversies persist regarding recommendation of IMNI in all patients with ALN metastasis. As a result, we need an accurate and effective procedure to detect subclinical metastases in IMN, in order to guide selection of high-risk population with IMN metastasis and best present the benefit of IMNI

### Toxicity of internal mammary lymph nodes irradiation

#### Dose to organ-at-risk in delivering internal mammary lymph nodes irradiation

One of major concerns about IMNI is the increased dose-volume of cardiac and pulmonary irradiation [12–14]. In the DBCG-IMN study, due to concern of the risk of radiation-induced heart disease, only patients with right-sided breast cancer were allocated to IMNI. In a dosimetric analysis of this study, mean heart dose was found to increase by a median of 4.8 Gy (0.9–8.7 Gy, *P* < 0.05) if IMNI was performed without dose-volume constraints in organs-at-risk [9, 15, 16]. Long term follow-up of randomized clinical trials has proven that irradiation exposure of the heart during breast cancer radiotherapy would increase the subsequent risk of heart disease [13, 17]. The dose response relationship established by Darby et al. suggested that the risk of ischemic heart disease increased by about 7% (95% CI, 3%–14%) for each 1 Gy increase in the mean dose of irradiation to the heart [18]. Similarly, Sardaro et al. estimated a 4% increase in the risk of heart disease for each 1 Gy increment in mean heart dose based on a large study of breast cancer patients treated with radiotherapy in Denmark and Sweden [19]. The results of MA.20 study also showed that patients in the RNI (including IMN) group had a higher rate of grade II or greater acute pneumonitis (1.2% vs. 0.2%, *P* = 0.01) and lymphedema (8.4% vs. 4.5%, *P* = 0.001) [5]. Further follow-up should be performed to evaluate the late side effects of IMNI.

Except for increase dose-volume of cardiac and pulmonary irradiation, synergistic adverse effect of IMNI and systemic therapy on normal tissue has also led to specific concern. About 20–25% of breast cancer patients had human epidermal growth factor receptor 2 (HER2) over-expressive tumors, which could benefit from trastuzumab with increase in DFS and OS [20, 21]. Similar to radiotherapy, trastuzumab is associated with increased cardiac toxicity. In clinical practice, adjuvant radiation therapy is usually administered in concurrent with trastuzumab, which raises concern about cardiac safety in delivering IMNI in left-sided patients treated with trastuzumab. Cao et al. has performed a series of studies to evaluate early cardiac toxicity associated with adjuvant radiotherapy of left-sided breast cancer with concurrent trastuzumab [22–24]. Result from these studies showed that concurrent trastuzumab and left-sided radiotherapy could be well tolerated in terms of early cardio-toxicity in patients with normal baseline cardiac function after adjuvant chemotherapy. In a prospective study of 106 breast cancer patients treated with concurrent trastuzumab-radiotherapy, Caussa et al. also reported similar result. Of these 106 patients, 88 patients (83%) received IMNI, which included 40 patients with left-sided breast cancer. After 28-month median follow-up, only six patients developed reversible ≥ grade II LVEF dysfunction [25]. Nevertheless, studies published by Cao et al. also reported association between increased low dose-volume, mean heart dose and increased acute left ventricular ejection fraction (LVEF) dysfunction, left ventricular diastolic dysfunction [23]. As a result, when IMNI was delivered concurrently with trastuzumab in left-sided patients, careful evaluation of baseline cardiac function before radiotherapy and strict dose-volume constraints in heart should be performed to guarantee cardiac safety.

#### Control of side effect

IMNI was associated with significant increase in dose-volume irradiation of heart and lung when outdated techniques were used [13, 18, 26]. With the development of modern techniques, irradiation dose to cardiac and pulmonary structures from breast radiotherapy have generally decreased during recent decades. The left-right difference in cardiac exposure to irradiation is less evident in case of IMNI through a separate anterior field [27]. Visualization of organs at risk (OARs) in the computerized tomography (CT) planning enables precise volumetric and geographical definition of clinical target volume and thus minimizes dose to the OARs [28]. Intensity-modulated radiotherapy (IMRT) has also shown promising results as a cardiac and pulmonary sparing technique for breast cancer patients in several reports [29, 30]. In addition, MacDonald et al. showed the great potential of proton radiotherapy in lowering dose to cardiac and pulmonary structures in treating chest wall and regional lymph nodes (including IMN) [31]. With decreasing in dose-volume of OARs irradiation, the risk of radiation-induced toxicity was also obviously reduced during recent decades. Højris et al. compared patients in Denmark who were treated during 1982–1990 with or without PMRT (including IMN) and found no increased risk of cardiovascular disease in patients received irradiation [32]. In the French IMN study, the grade III-IV late side effects of irradiation were roughly of the same order of magnitude (3.1%, 21/672 vs. 2.3%, 15/662) and no significant increase in late cardiac events (2.2%, 15/672 vs. 1.7%, 11/662) observed in the IMNI group [8].

Individual positioning is a feasible approach through which to reduce radiation dose to the OARs [33, 34]. The prone positioning has been reported to significantly reduce the lung dose and the heart exposure in breast cancer patients [33–36]. For patients with unfavorable anatomy, breath hold techniques and the additional heart block may be useful to spare irradiation of the cardiac or pulmonary structures [32, 37]. Deep inspiration breath-hold (DIBH) have shown a promise in reducing heart doses without compromising target volume or increasing contralateral breast dose. DIBH could increase spatial separation between the heart and the target volume, which results in a decreased exposure volume of the heart within the tangential fields [38]. Furthermore, risks of radiotherapy induced major coronary events could be reduced by targeting baseline cardiac risk factors (cholesterol, smoking, hypertension), by modification of lifestyle modification and pharmacological treatment [39].

### Potential strategies to tailor internal mammary lymph node irradiation

Although the 2016 NCCN Breast Cancer Clinical Practice Guidelines recommends IMNI for patients with ≥ 4 positive ALNs, and strongly considers IMN for patients with 1–3 positive ALNs, there is lack of evidence to establish the risk of IMN involvement [40]. Studies of extended radical mastectomy reported that 38.3% (36.8%–46.2%) of patients with ≥ 4 positive ALNs, 19.6% (18.8%–26.7%) of patients with 1–3 positive ALNs, and 9.2% (4.4%–16.8%) of patients with negative ALNs had IMN metastases. The fact was that, negative IMN was found in about 60% of patients with ≥ 4 positive ALNs and positive IMN was found in about 9% patients with negative ALNs [41–43]. The population of overlapped risk remains a clinical dilemma. In clinical practice, imaging techniques, such as positron emission tomography/computed tomography, ultrasound and magnetic resonance imaging, could usually detect lesions ≥ 5 mm [40], but the metastases lesions in IMN is too small (median 4 mm, range 0.7–11 mm) to be detected in general [44]. The results of extended radical mastectomy showed that patients with following conditions had high-risk of IMNs metastasis (> 20%): (1) ≥ 4 positive ALNs, (2) medial tumor and positive ALNs, (3) T3 tumor and younger than 35-year-old, (4) T2 tumor and positive ALNs, (5) T2 tumor and medial tumor [43]. The frequency of IMN recurrence is low (mean 0.9%, range 0.1–1.5%) [45–47] and ER/PR status is a risk factor for DFS of IMN recurrence [47]. However, all these methods are not enough to guide the individualized IMNI in the clinical practice. Therefore, a more accurate technique is required to better evaluate the pathological status of IMN and to guide IMNI.

The study by Veronesi et al. found that IMNI could improve the survival in patients with metastases in IMN identified by internal mammary lymph node biopsy. In this large series of 663 patients, 68 (10.3%, 68/663) patients received IMNI for histologically proven IMN metastases. IMNI was effective yielded a 5-year OS of 95% to compare with IMN negative without IMNI [48].

Currently, internal mammary sentinel lymph node biopsy (IM-SLNB) via intercostal space could be an accurate technique to guide personalized IMNI and minimize invasive staging in IMN [40, 49, 50]. Even though breast cancer staging has incorporated IM-SLNB concept since the 6th edition of the American Joint Committee on Cancer, IM-SLNB has not been performed routinely in the clinical practice [51]. The studies of IM-SLNB showed that the success rate of IM-SLNB has reached 60%–100% with minimal or no prolong in the operative time, but the visualization rate of IM-SLN was low [44, 52–54], which has been the restriction for both clinical study and daily practice of IM-SLNB. Now, a modified radiotracer injection technique was established based on the IM-SLN lymphatic drainage pattern and could significantly improve the IM-SLN detection rate from 15.5% to 71% (*P* < 0.001) [55]. The accuracy of the modified injection technique and the IM-SLN lymphatic drainage pattern has been validated by our team [56].

Up to now, 313 patients with breast cancer received IM-SLNB guided by the modified radiotracer injection technique. The overall visualization rate of IM-SLN detected by preoperative lymphoscintigraphy and gamma probe was 70.9% (327/461). The success rate of IM-SLNB was 97.1% (304/313). In patients who performed IM-SLNB successfully, a total of 585 lymph nodes were removed, the median number of IM-SLNs was 2 (range 1–4 nodes). The IM-SLNs were located in the first (5.5%, 32/585), second (46.3%, 271/585), third (40.5%, 237/585) and forth (7.7%, 45/585) intercostal space. The positive IM-SLNs were located in the first (1.6%, 1/64), second (56.3%, 36/64), and the third (42.1%, 27/64) intercostal space. Our data showed that the IM-SLN involvement rate was 8.3% (18/218) in patient with ALN negative and 18.6% (16/86) in ALN positive patients. The significant meaning of IM-SLNB, is anticipated that, to detect the sub-clinical involvement of IMN, and thus to guide IMNI in patients who might not have been recommended for IMNI, as well as to guide not to give IMNI in patients who might been recommended for IMNI by current NCCN guideline. Our current data shows that, in patients with ≥4 positive ALNs, IMNI could be avoided in 57.1% cases (20/35) with negative IM-SLN. In patients with 1–3 positive ALNs, IMNI could be avoided in 90.6% cases (115/127) with negative IM-SLN. Our study is undergoing and needs a long-term follow-up to evaluate the benefit and the late side-effect of this method. The indication of IM-SLNB should be identified from person to person in the future (eg. ALN positive, IMN metastasis with ALN negative, or medial tumor). Currently, we are designing a clinical trial to explore whether IMNI following IM-SLNB should be performed to control the local recurrence in patients with IM-SLN metastasis (Trial registration ID: NCT03024463). We hope the clinical trial will have a good result to guide the clinical practice of IMNI.

## CONCLUSIONS

As the part of the regional nodes irradiation for breast cancer radiotherapy, IMNI could partly contribute to a reduction in recurrence and improved survival in some population. The balance between the technical complexities and toxicities of IMNI and relative low frequency of clinically detected IMN involvement has posed therapeutic dilemma even if the NCCN guideline has upgraded its recommendation. An effective and accurate method is needed to individualize the patient selection of IMNI so that the benefit of IMNI can be performed in patients with IMN metastasis. IM-SLNB would make it possible which is a minimally invasive technique to evaluate the metastasis status in IMN, improve the nodal staging in IMN chain, and would be used to guide personalized IMNI.

## References

[1] Veronesi U, Cascinelli N, Greco M, Bufalino R, Morabito A, Galluzzo D, Conti R, De Lellis R, Delle Donne V, Piotti P (1985). Prognosis of breast cancer patients after mastectomy and dissection of internal mammary nodes. Ann Surg.

[2] Cody HS, Urban JA (1995). Internal mammary node status: a major prognosticator in axillary node-negative breast cancer. Ann Surg Oncol.

[3] Poortmans P (2014). Postmastectomy radiation in breast cancer with one to three involved lymph nodes: ending the debate. Lancet.

[4] McGale P, Taylor C, Correa C, Cutter D, Duane F, Ewertz M, Gray R, Mannu G, Peto R, Whelan T, Wang Y, Wang Z, EBCTCG (Early Breast Cancer Trialists’ Collaborative Group) (2014). Effect of radiotherapy after mastectomy and axillary surgery on 10-year recurrence and 20-year breast cancer mortality: meta-analysis of individual patient data for 8135 women in 22 randomised trials. Lancet.

[5] Whelan TJ, Olivotto IA, Parulekar WR, Ackerman I, Chua BH, Nabid A, Vallis KA, White JR, Rousseau P, Fortin A, Pierce LJ, Manchul L, Chafe S (2015). Regional nodal irradiation in early-stage breast cancer. N Engl J Med.

[6] Poortmans PM, Collette S, Kirkove C, Van Limbergen E, Budach V, Struikmans H, Collette L, Fourquet A, Maingon P, Valli M, De Winter K, Marnitz S, Barillot I (2015). Internal mammary and medial supraclavicular irradiation in breast cancer. N Engl J Med.

[7] Stemmer SM, Rizel S, Hardan I, Adamo A, Neumann A, Goffman J, Brenner HJ, Pfeffer MR (2003). The role of irradiation of the internal mammary lymph nodes in high-risk stage II to IIIA breast cancer patients after high-dose chemotherapy: a prospective sequential nonrandomized study. J Clin Oncol.

[8] Hennequin C, Bossard N, Servagi-Vernat S, Maingon P, Dubois JB, Datchary J, Carrie C, Roullet B, Suchaud JP, Teissier E, Lucardi A, Gerard JP, Belot A (2013). Ten-year survival results of a randomized trial of irradiation of internal mammary nodes after mastectomy. Int J Radiat Oncol Biol Phys.

[9] Thorsen LB, Offersen BV, Danø H, Berg M, Jensen I, Pedersen AN, Zimmermann SJ, Brodersen HJ, Overgaard M, Overgaard J (2016). DBCG-IMN: A population-based cohort study on the effect of internal mammary node irradiation in early node-positive breast cancer. J Clin Oncol.

[10] Budach W, Bölke E, Kammers K, Gerber PA, Nestle-Krämling C, Matuschek C (2013). Adjuvant radiation therapy of regional lymph nodes in breast cancer - a meta-analysis of randomized trials- an update. Radiat Oncol.

[11] Recht A, Comen EA, Fine RE, Fleming GF, Hardenbergh PH, Ho AY, Hudis CA, Hwang ES, Kirshner JJ, Morrow M, Salerno KE, Sledge GW, Solin LJ (2016). Postmastectomy Radiotherapy: An American Society of Clinical Oncology, American Society for Radiation Oncology, and Society of Surgical Oncology Focused Guideline Update. J Clin Oncol.

[12] Taylor CW, Wang Z, Macaulay E, Jagsi R, Duane F, Darby SC (2015). Exposure of the heart in breast cancer radiation therapy: a systematic review of heart doses published during 2003 to 2013. Int J Radiat Oncol Biol Phys.

[13] Leung HW, Chan AL, Muo CH (2016). Late cardiac morbidity of adjuvant radiotherapy for early breast cancer - A population-based study. J Cardiol.

[14] Choi J, Kim YB, Shin KH, Ahn SJ, Lee HS, Park W, Kim SS, Kim JH, Lee KC, Kim DW, Suh HS, Park KR, Shin HS (2016). Radiation Pneumonitis in Association with Internal Mammary Node Irradiation in Breast Cancer Patients: An Ancillary Result from the KROG 08–06 Study. J Breast Cancer.

[15] Thorsen LB, Thomsen MS, Overgaard M, Overgaard J, Offersen BV, Danish Breast Cancer Cooperative Group Radiotherapy Committee (2013). Quality assurance of conventional non-CT-based internal mammary lymph node irradiation in a prospective Danish Breast Cancer Cooperative Group trial: the DBCG-IMN study. Acta Oncol.

[16] Thorsen LB, Thomsen MS, Berg M, Jensen I, Josipovic M, Overgaard M, Overgaard J, Skogholt P, Offersen BV, Danish Breast Cancer Cooperative Group (2014). Radiotherapy committee CT-planned internal mammary node radiotherapy in the DBCG-IMN study: benefit versus potentially harmful effects. Acta Oncol.

[17] Sardar P, Kundu A, Chatterjee S, Nohria A, Nairooz R, Bangalore S, Mukherjee D, Aronow WS, Lavie CJ (2017). Long-term cardiovascular mortality after radiotherapy for breast cancer: A systematic review and meta-analysis. Clin Cardiol.

[18] Darby SC, Ewertz M, McGale P, Bennet AM, Blom-Goldman U, Brønnum D, Correa C, Cutter D, Gagliardi G, Gigante B, Jensen MB, Nisbet A, Peto R (2013). Risk of ischemic heart disease in women after radiotherapy for breast cancer. N Engl J Med.

[19] Sardaro A, Petruzzelli MF, D’Errico MP, Grimaldi L, Pili G, Portaluri M (2012). Radiation-induced cardiac damage in early left breast cancer patients: risk factors, biological mechanisms, radiobiology, and dosimetric constraints. Radiother Oncol.

[20] Piccart-Gebhart MJ, Procter M, Leyland-Jones B, Goldhirsch A, Untch M, Smith I, Gianni L, Baselga J, Bell R, Jackisch C, Cameron D, Dowsett M, Barrios CH (2005). Trastuzumab after adjuvant chemotherapy in HER2-positive breast cancer. N Engl J Med.

[21] Viani GA, Afonso SL, Stefano EJ, De Fendi LI, Soares FV (2007). Adjuvant trastuzumab in the treatment of her-2-positive early breast cancer: a meta-analysis of published randomized trials. BMC Cancer.

[22] Cao L, Cai G, Chang C, Miao AY, Yu XL, Yang ZZ, Ma JL, Zhang Q, Wu J, Guo XM, Chen JY (2015). Diastolic dysfunction occurs early in HER2-positive breast cancer patients treated concurrently with radiation therapy and trastuzumab. Oncologist.

[23] Cao L, Cai G, Chang C, Yang ZZ, Feng Y, Yu XL, Ma JL, Wu J, Guo XM, Chen JY (2015). Early cardiac toxicity following adjuvant radiotherapy of left-sided breast cancer with or without concurrent trastuzumab. Oncotarget.

[24] Cao L, Hu WG, Kirova YM, Yang ZZ, Cai G, Yu XL, Ma JL, Guo XM, Shao ZM, Chen JY (2014). Potential impact of cardiac dose-volume on acute cardiac toxicity following concurrent trastuzumab and radiotherapy. Cancer Radiother.

[25] Caussa L, Kirova YM, Gault N, Pierga JY, Savignoni A, Campana F, Dendale R, Fourquet A, Bollet MA (2011). The acute skin and heart toxicity of a concurrent association of trastuzumab and locoregional breast radiotherapy including internal mammary chain: a single-institution study. Eur J Cancer.

[26] Taylor CW, McGale P, Povall JM, Thomas E, Kumar S, Dodwell D, Darby SC (2009). Estimating cardiac exposure from breast cancer radiotherapy in clinical practice. Int J Radiat Oncol Biol Phys.

[27] Shapiro CL, Hardenbergh PH, Gelman R, Blanks D, Hauptman P, Recht A, Hayes DF, Harris J, Henderson IC (1998). Cardiac effects of adjuvant doxorubicin and radiation therapy in breast cancer patients. J Clin Oncol.

[28] Nielsen MH, Berg M, Pedersen AN, Andersen K, Glavicic V, Jakobsen EH, Jensen I, Josipovic M, Lorenzen EL, Nielsen HM, Stenbygaard L, Thomsen MS, Vallentin S (2013). Delineation of target volumes and organs at risk in adjuvant radiotherapy of early breast cancer: National guidelines and contouring atlas by the Danish Breast Cancer Cooperative Group. Acta Oncol.

[29] Giraud P, Yorke E, Jiang S, Simon L, Rosenzweig K, Mageras G (2006). Reduction of organ motion effects in IMRT and conformal 3D radiation delivery by using gating and tracking techniques. Cancer Radiother.

[30] Shah C, Badiyan S, Berry S, Khan AJ, Goyal S, Schulte K, Nanavati A, Lynch M, Vicini FA (2014). Cardiac dose sparing and avoidance techniques in breast cancer radiotherapy. Radiother Oncol.

[31] MacDonald SM, Jimenez R, Paetzold P, Adams J, Beatty J, DeLaney TF, Kooy H, Taghian AG, Lu HM (2013). Proton radiotherapy for chest wall and regional lymphatic radiation; dose comparisons and treatment delivery. Radiat Oncol.

[32] Højris I, Overgaard M, Christensen JJ, Overgaard J (1999). Morbidity and mortality of ischaemic heart disease in high-risk breast-cancer patients after adjuvant postmastectomy systemic treatment with or without radiotherapy: analysis of DBCG 82b and 82c randomised trials. Radiotherapy Committee of the Danish Breast Cancer Cooperative Group. Lancet.

[33] Formenti SC, Gidea-Addeo D, Goldberg JD, Roses DF, Guth A, Rosenstein BS, DeWyngaert KJ (2007). Phase I-II trial of prone accelerated intensity modulated radiation therapy to the breast to optimally spare normal tissue. J Clin Oncol.

[34] Formenti SC, DeWyngaert JK, Jozsef G, Goldberg JD (2012). Prone vs supine positioning for breast cancer radiotherapy. JAMA.

[35] Lymberis SC, deWyngaert JK, Parhar P, Chhabra AM, Fenton-Kerimian M, Chang J, Hochman T, Guth A, Roses D, Goldberg JD, Formenti SC (2012). Prospective assessment of optimal individual position (prone versus supine) for breast radiotherapy: Volumetric and dosimetric correlations in 100 patients. Int J Radiat Oncol Biol Phys.

[36] Varga Z (2014). Individualized positioning for maximum heart protection during breast irradiation. Acta Oncol.

[37] Ma J, Li J, Xie J, Chen J, Zhu C, Cai G, Zhang Z, Guo X, Chen J (2013). Post mastectomy linac IMRT irradiation of chest wall and regional nodes: dosimetry data and acute toxicities. Radiat Oncol.

[38] Fung E, Hendry J (2013). External beam radiotherapy (EBRT) techniques used in breast cancer treatment to reduce cardiac exposure. Radiography.

[39] Brenner DJ, Shuryak I, Jozsef G, Dewyngaert KJ, Formenti SC (2014). Risk and risk reduction of major coronary events associated with contemporary breast radiotherapy. JAMA Intern Med.

[40] Cong BB, Qiu PF, Wang YS (2014). Internal mammary sentinel lymph node biopsy: minimally invasive staging and tailored internal mammary radiotherapy. Ann Surg Oncol.

[41] Veronesi U, Valagussa P (1981). Inefficacy of internal mammary nodes dissection in breast cancer surgery. Cancer.

[42] Noguchi M, Ohta N, Thomas M, Kitagawa H, Miyazaki I (1993). Risk of internal mammary lymph node metastases and its prognostic value in breast cancer patients. J Surg Oncol.

[43] Huang O, Wang L, Shen K, Lin H, Hu Z, Liu G, Wu J, Lu J, Shao Z, Han Q, Shen Z (2008). Breast cancer subpopulation with high risk of internal mammary lymph nodes metastasis: analysis of 2,269 Chinese breast cancer patients treated with extended radical mastectomy. Breast Cancer Res Treat.

[44] Caudle AS, Yi M, Hoffman KE, Mittendorf EA, Babiera GV, Hwang RF, Meric-Bernstam F, Sahin AA, Hunt KK (2014). Impact of identification of internal mammary sentinel lymph node metastasis in breast cancer patients. Ann Surg Oncol.

[45] Dellapasqua S, Bagnardi V, Balduzzi A, Iorfida M, Rotmensz N, Santillo B, Viale G, Ghisini R, Veronesi P, Luini A, Morra A, Goldhirsch A, Colleoni M (2014). Outcomes of patients with breast cancer who present with ipsilateral supraclavicular or internal mammary lymph node metastases. Clin Breast Cancer.

[46] Sutton EJ, Watson EJ, Gibbons G, Goldman DA, Moskowitz CS, Jochelson MS, Dershaw DD, Morris EA (2015). Incidence of internal mammary lymph nodes with silicone breast implants at MR imaging after oncoplastic surgery. Radiology.

[47] Chen L, Gu Y, Leaw S, Wang Z, Wang P, Hu X, Chen J, Lu J, Shao Z (2010). Internal mammary lymph node recurrence: rare but characteristic metastasis site in breast cancer. BMC Cancer.

[48] Veronesi U, Arnone P, Veronesi P, Galimberti V, Luini A, Rotmensz N, Botteri E, Ivaldi GB, Leonardi MC, Viale G, Sagona A, Paganelli G, Panzeri R (2008). The value of radiotherapy on metastatic internal mammary nodes in breast cancer. Results on a large series. Ann Oncol.

[49] Cong BB, Cao XS, Qiu PF, Yu JM, Wang YS (2017). Internal mammary sentinel lymph node biopsy: An effective way to search benefit patients and guide internal mammary chain irradiation. Breast.

[50] Asadi M, Krag D (2016). Internal mammary sentinel lymph node biopsy in clinical practice. Int J Surg.

[51] Connolly JL (2006). Changes and problematic areas in interpretation of the AJCC Cancer Staging Manual, 6th Edition, for breast cancer. Arch Pathol Lab Med.

[52] Heuts EM, van der Ent FW, von Meyenfeldt MF, Voogd AC (2009). Internal mammary lymph drainage and sentinel node biopsy in breast cancer - A study on 1008 patients. Eur J Surg Oncol.

[53] Postma EL, van Wieringen S, Hobbelink MG, Verkooijen HM, van den Bongard HJ, Borel Rinkes IH, Witkamp AJ (2012). Sentinel lymph node biopsy of the internal mammary chain in breast cancer. Breast Cancer Res Treat.

[54] Gnerlich JL, Barreto-Andrade JC, Czechura T, John JR, Turk MA, Kennedy TJ, Winchester DJ (2014). Accurate staging with internal mammary chain sentinel node biopsy for breast cancer. Ann Surg Oncol.

[55] Qiu PF, Cong BB, Zhao RR, Yang GR, Liu YB, Chen P, Wang YS (2015). Internal mammary sentinel lymph node biopsy with modified injection technique: high visualization rate and accurate staging. Medicine (Baltimore).

[56] Cong BB, Qiu PF, Liu YB, Zhao T, Chen P, Cao XS, Wang CJ, Zhang ZP, Sun X, Yu JM, Wang YS (2016). Validation study for the hypothesis of internal mammary sentinel lymph node lymphatic drainage in breast cancer. Oncotarget.

